# Evaluation of the Operator Protection Factors Offered by Positive Pressure Air Suits against Airborne Microbiological Challenge 

**DOI:** 10.3390/v4081202

**Published:** 2012-08-07

**Authors:** Jackie A. Steward, Mark S. Lever

**Affiliations:** Defence Science and Technology Laboratory (Dstl), Porton Down, Salisbury, Wiltshire, SP4 0JQ, UK

**Keywords:** Risk Group 4, positive pressure suits, aerosol microbiological challenge, OPF

## Abstract

Laboratories throughout the world that perform work with Risk Group 4 Pathogens generally adopt one of two approaches within BSL-4 environments: either the use of positive pressure air-fed suits or using Class III microbiological safety cabinets and isolators for animal work. Within the UK at present, all laboratories working with Risk Group 4 agents adopt the use of Class III microbiological safety cabinet lines and isolators. Operator protection factors for the use of microbiological safety cabinets and isolators are available however; there is limited published data on the operator protection factors afforded by the use of positive pressure suits. This study evaluated the operator protection factors provided by positive pressure air suits against a realistic airborne microbiological challenge. The suits were tested, both intact and with their integrity compromised, on an animated mannequin within a stainless steel exposure chamber. The suits gave operator protection in all tests with an intact suit and with a cut in the leg. When compromised by a cut in the glove, a very small ingress of the challenge was seen as far as the wrist. This is likely to be due to the low airflow in the gloves of the suit. In all cases no microbiological penetration of the respiratory tract was observed. These data provide evidence on which to base safety protocols for use of positive pressure suits within high containment laboratories.

## 1. Introduction

There are two main operational systems for conducting BSL-4 work throughout the world; manipulations within a totally enclosed cabinet line systems and a suited system, where workers wear a protective suit while manipulating Risk Group 4 Pathogens (RG4) in combination with other engineered controls such as class II cabinets or laminar flow. Cabinet line systems protect the worker and the immediate environment through a combination of good microbiological techniques and the use of rigid animal isolators (for high containment animal work) or a cabinet line consisting of Class III microbiological safety cabinets (for *in vitro* work). Positive pressure air-fed suits are designed for positive pressure to prevent contamination to the wearer even in the event of damage to the suit.

Over the last 30 years within the UK, cabinet line systems have been routinely used and the Defence Science and Technology Laboratory (Dstl) Porton Down, UK has been working with Advisory Committee on Dangerous Pathogens (ACDP) containment level 4 (equivalent to BSL-4) rigid half suit isolators and microbiological safety cabinet line (CL III cabinets) for 10 years with Risk Group 4 pathogens [1]. Throughout the US, Canada and Europe the use of positive pressure suits have been used.

There are advantages and disadvantages associated with each system, and is very much dependent on the tasks to be performed. For example, if the majority of work performed is the routine handling of diagnostic samples, then cabinet lines may offer a simpler and more efficient system within which to work. Alternatively, if the work involves large non-human primates housed within high containment conditions or the use of large items of equipment, then suited systems offer a more appropriate alternative. Unlike microbiological safety cabinets, there is limited published data on the operator protection factor (OPF) afforded by positive pressure suits [2]; therefore, tests were devised by the Physical Sciences Department at Dstl to evaluate these suits. The operator protection factor is defined as the ratio of exposure to airborne contamination generated on the open bench to the exposure resulting from the same disposal of airborne contamination generated within the cabinet. When tested in accordance with the British Standard all cabinets in use should have an operator protection factor of at least 1.0 × 10^5^. The authors are unaware of any equivalent standard applicable to positive pressure suits.

The aim of this study was to assess the operator protection factor of positive pressure suits against an airborne microbiological challenge.

## 2. Material and Methods

### 2.1. Preparation of Challenge Material

*Bacillus atrophaeus* was used as the microbial challenge for the suits. *B. atrophaeus* was obtained from the Defence Science and Technology Laboratory (Dstl Porton Down, Salisbury, Wiltshire, UK) culture collection. The challenge inoculum for the spray was prepared as described previously [3].

### 2.2. Mannequin

**Figure 1:**
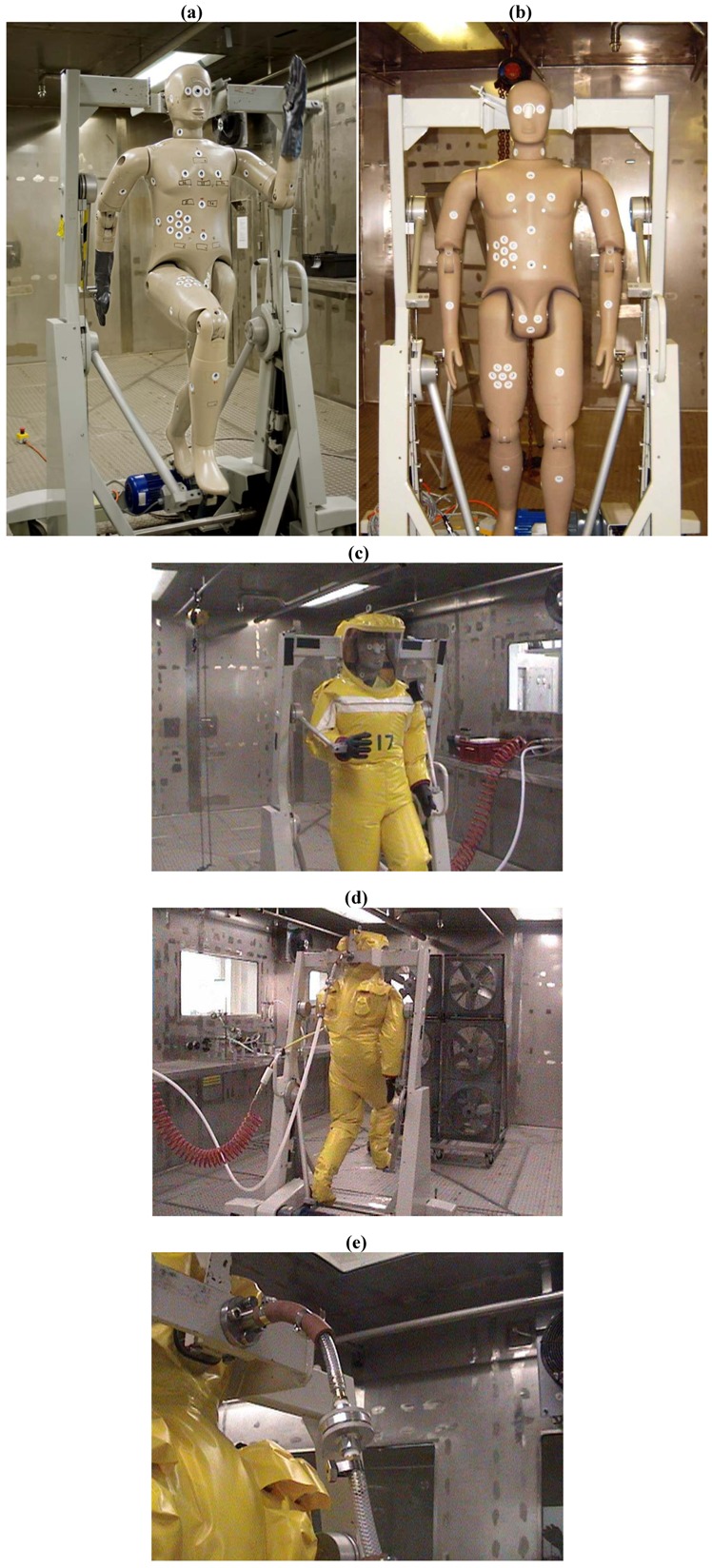
(**a**,**b**) Animated mannequin within the stainless steel exposure chamber. (**c**) Front view of mannequin dressed in surgical scrubs and the Respirex Simplair suit. (**d**) Rear view of suited mannequin showing fans within the exposure chamber. The red coiled pipe is the supply air and the white pipe is the breathing air supply. (**e**) Close-up view of the breathing air supply pipe with GF/A filter.

All tests were carried out using an animated mannequin (Figure 1a,b). The mannequin was articulated at the shoulder, elbow, hip and knee joints, and movement being restricted to the vertical plane only. The hands and feet of the mannequin are attached to a motor-driven pulley system. Adjustment of this pulley system allows the mannequin to simulate 4 degrees of motion between both normal walking and an exaggerated marching motion. The extent of limb movement is governed by the relative radii of the pulleys involved. The cycle time was adjustable and was set to a walking speed of approximately 4 mph.

### 2.3. Suit Tests

There are limited manufacturers of positive pressure suits. The Respirex Simplair suit was chosen to test. This suit was chosen as it is from a UK-based company, which would make it easier for regular supplies. The suits were tested both intact and with their integrity compromised. The mannequin was dressed in surgical scrubs and the positive pressure suit (Figure 1c–e).

All suit tests (Table 1) were carried out within a stainless steel exposure chamber using an animated mannequin. Integrity testing of the suits was performed prior to the bacterial challenge by positive pressure hold testing over 30 minutes.

A 100 mL suspension of *B. atrophaeus* (formally *Bacillus subtilis* var. *niger* and *Bacillus globii*) at a concentration of 1 × 10^7^ cfu m^−3^ was released using a two-fluid atomizer into the chamber to produce an aerosol with a mass median diameter of approximately 1 micron. This was repeated every 30 minutes to maintain the concentration within the chamber. Two open face glass microfiber (GF/A) filters were used to measure the challenge concentration. The particle size and chamber concentration were monitored in real time throughout the experiment using an Aerodynamic Particle Sizer (APS). This allowed the challenge to the suit to be calculated from the chamber concentration/time profile.

**Figure 2:**
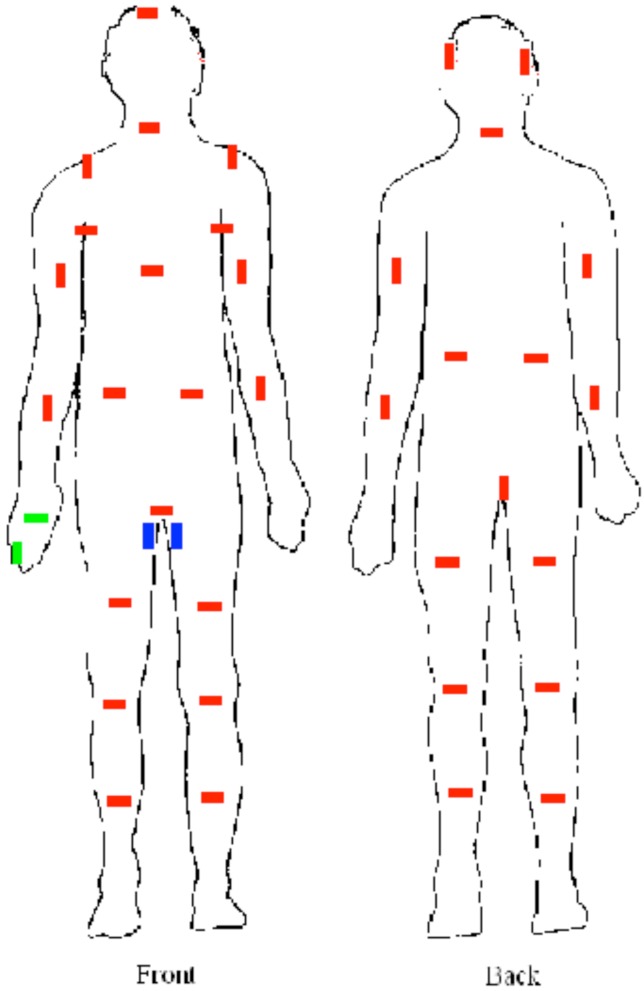
Sampling positions on mannequin.

At the end of the exposure time of 2 hours, swabs were taken from a number of skin level depositions at various locations on the mannequin’s body (Figure 2). The swabs were plated onto L‑agar for enumeration of viable *B. atrophaeus* organisms.

**Table 1:** Summary of tests performed on a number of suits.

Suit ID	Test conditions
1	Intact
2	Intact
3	2 cm cut in Index finger right hand glove (Figure 3) (2 extra swabs on hand—shown in green on Figure 2)
4	2 cm cut in Index finger right hand glove (Figure 3) (2 extra swabs on hand—shown in green on Figure 2)
5	10 cm cut down right hand inside leg from groin (Figure 4) (2 extra swabs on upper inside leg—shown in blue on Figure 2)

### 2.4. Sampling

The mannequin was dressed in the Respirex Simplair suit on top of surgical scrubs. The air feed hose was connected to the suit and the air flow was set to 420 litres per minute (lpm). The mannequin was set in motion at a low level of movement and slow speed to simulate typical movements of personnel wearing the suits. Throughout the experiment the mannequin moved for 20 minutes and then rested for 20 minutes for a total of 2 hours. A bank of six fans (Figure 1d), positioned 2.5 m upwind of the mannequin, was started to generate a wind speed of 5 ms^−1^ to maintain a constant challenge to the suit and to ensure even mixing of the challenge within the chamber.

Sampling tapes were placed at various locations on the mannequin (Figure 2). Tapes were also attached to the frame to provide an additional indication of the challenge to the suit. An inline filter holder containing a GF/A filter was attached to the breath sampling port at the rear of the head of the mannequin and was connected to a pump set at a flow rate of 50 lpm to enable monitoring of *B. atrophaeus* penetration of the respiratory tract (Figure 1e).

At the end of the spray sedimentation plates were placed around the mannequin. Fresh sampling tape was also placed on the frame of the mannequin. The clothing was removed carefully from the mannequin to minimise re-aerosolisation and single swab samples (40 cm^2^) were taken from the sampling tapes on the mannequin and frame. The chamber was decontaminated by formaldehyde fumigation after each suit test. All swabs and collector samples were diluted in L-broth and plated out on L-agar then incubated at 37 °C for enumeration of viable *B. atrophaeus* colonies.

Results were calculated for each of the five suits tested. The effects of re-aerosolisation and background contamination were removed (based on the sedimentation plates).

## 3. Results

### 3.1. Operator Protection Factor

The results from tests on suit 1 have been omitted from Table 2 as it was known to be contaminated during the experiment; however, the tests were repeated on another intact suit (suit 2). *B. atrophaeus* growth from swabs was found on the palm of the mannequin during tests of suit 3, and the rear lower right arm and palm of the mannequin during suit 4 tests. No ingress of spores on any sampling position on the mannequin was detected during tests on both suits 2 and 5. No *B. atrophaeus* penetration of the respiratory tract was observed in any of the suits tested.

**Table 2:** Summary of cfu/swab per body position. Only those body positions that proved positive are shown in the table. All other swabs were negative for viable *B. atrophaeus* colonies.

Position	Suit 2 (intact suit)	Suit 3 (cut glove)	Suit 4 (cut glove)	Suit 5 (cut leg)
**Rear lower right arm**	-	-	12	-
**Palm**	-	48	9	-
**Groin**	-	-	-	-

- No growth observed on agar plates.

### 3.2. Intact Suit System

As previously stated suit 1 was removed from the study. Suit 2 results demonstrated that whilst the suit remained intact it provided protection (including respiratory protection) against a microbiological challenge of 1.0 × 10^7 ^*B. atrophaeus* spores.

### 3.3. Compromised Suit System — Cut in Glove

Tests performed on a compromised suit (cut in glove of suit 3) indicated that there was penetration of *B. atrophaeus* spores into the glove, with some spores deposited on the palm of the mannequin. For suit 4, with a similar cut in the glove, penetration was again observed with spores deposited on the palm and lower arm of the mannequin. For both compromised suits no *B. atrophaeus* penetration of the respiratory tract was observed.

**Figure 3:**
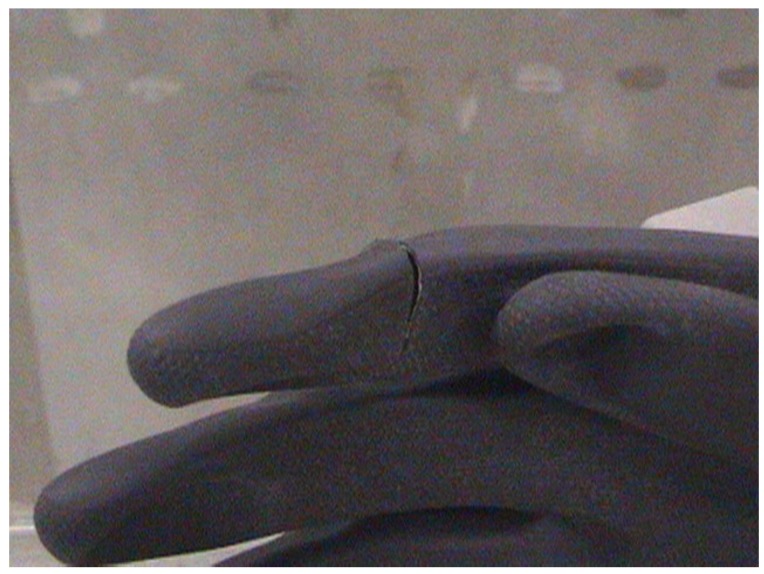
An example of the cut made in the glove of suits 3 and 4.

### 3.4. Compromised Suit System — Cut in Leg

No *B. atrophaeus* spores were detected on any sample position including the respiratory tract of the mannequin when the suit was compromised by a cut on the leg.

**Figure 4:**
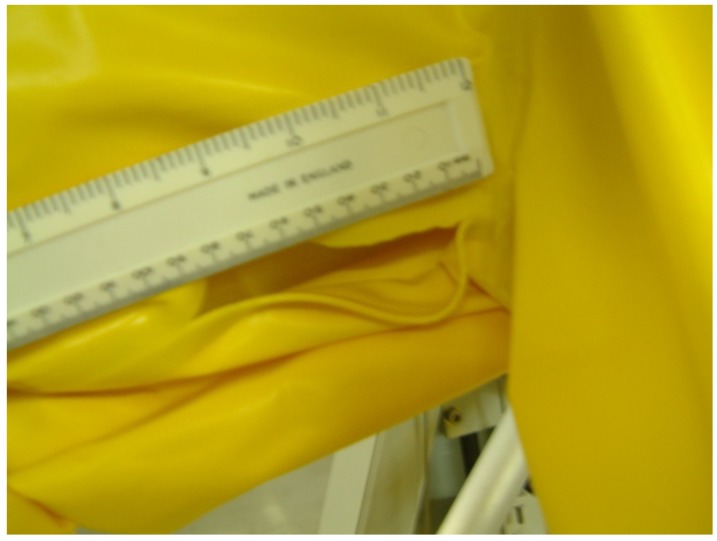
A cut in the inner thigh area of the leg of suit 5.

## 4. Discussion

The objective of this study was to assess the protection afforded by full positive-pressure suits against an aerosol challenge under normal and realistic accident scenarios. There have been few studies performed previously to assess the operator protection factors of suits with which to compare the current data. The operator protection tests demonstrated that an exceptionally high level of protection was provided by the suited system under normal working conditions. Tests demonstrated that the OPF was significantly higher than the minimum required OPF of 10^5^, recommended for microbiological safety cabinets. Such levels of protection are equivalent to those reported previously within a cabinet line system [1].

When the integrity of the suit was compromised by a small cut in the glove the protection of the suit was reduced by approximately ten-fold, however this was still above the minimum recommended protection level (10^5^). It is likely that the ingress seen at the glove is due to the lowered airflow in the gloves of the suit, due to constriction of airflow under the suit at the wrists. As long as the suit remained at positive pressure, relative to the external environment however, protection levels greater than 10^5^ were maintained.

When compromised by a cut in the leg the positive pressure inside the suit prevented ingress of the challenge micro-organisms. It is likely that the air-flow within the leg of the suit was greater than that seen at the wrist and was sufficient to maintain positive pressure to prevent ingress of micro‑organisms. In all of the suits tested there are no filters or air pipes positioned near any of the compromised areas. In all cases, no *B. atrophaeus* penetration of the respiratory tract was observed.

The operator protection factor provided by full positive pressure suits in this study was comparable to the protection factors calculated for a rigid half-suited isolator system tested under similar accident scenarios (in-house data). It was not possible to test the protection of the suits under loss of positive pressure in this experimental set-up as the respiratory tract sampling air caused a negative pressure within the suit once supply air had been turned-off.

There is very little data published previously on protection factors of positive pressure suits. Kumin *et al.* evaluated three different types of BSL-4 suits for their material compatibility against decontamination disinfectants, protection factors against chemicals and comfort for users [2]. Chemical protection factor tests included simulating movement whilst in the laboratory and chemical protection of intact suits. Direct comparison between the current study and that of Kumin *et al.* however, is difficult, as the current study looked at protection against biological aerosols whereas Kumin *et al.* assessed chemical protection against VX gas.

The tests in this current study used an aerosol microbiological challenge to measure the protective efficacy of the suited system. Published aerosol infective doses for Ebola virus in rhesus monkeys are as low as 400 plaque-forming units (pfu) [4,5] and Marburg virus has been shown to be lethal by the aerosol route in rhesus monkeys [6], grivets [7], and has an aerosol infective dose of less than 10 tissue culture infective doses (TCID_50_) in marmosets (manuscript submitted) and mice [8]. It was shown that 1 pfu was approximately equivalent to 25–30 virions [9]. Therefore, the infectious dose for rhesus monkeys could be between 10,000–12,000 virions. Filovirus-infected non-human primates represent an appropriate animal model for predicting human infectious doses of filoviruses [10], therefore the aerosol-infectious dose for humans may be reasonably assumed to be similar. In a monodispersed aerosol (of size range 1–3 µM) of bacterial spores (as used in the current study), each particle would usually contain one bacterium. A suspension of virus at an equivalent starting concentration would generate particles each reasonably expected to contain a number of virions (up to ten-fold more based on average filovirus dimensions of approximately 80 nm × 800 nm). Based on these theoretical calculations therefore, a damaged suit with a ten-fold reduction in protection, challenged with an aerosol of approximately 1.0 × 10^8^ virions, would theoretically let in approximately 100 virions. This is equivalent to approximately 4 pfu therefore even in the worst case scenario this would be approximately 100 times less than the theoretical infectious dose for rhesus monkeys (and by extrapolation humans).

Many laboratory-acquired infections have been reported in the scientific literature [11,12]. Historically, procedures such as centrifugation or needle stick injuries are the main cause of laboratory-acquired infections [13]. Clearly, for needle stick injuries, positive pressure suits offer no greater protection than safety cabinets. In summary, positive pressure suited systems provide exceptionally good operator protection, even under accident scenarios, and potentially provide greater flexibility and ergonomic benefits to the user.

## Notes

© Crown Copyright. Dstl, 2012.
